# Total Breast Reconstruction with Lipofilling after Traditional Mastectomy without the Use of Tissue Expanders

**DOI:** 10.1097/PRS.0000000000010252

**Published:** 2023-02-14

**Authors:** Pauliina Homsy, Anna Höckerstedt, Katja Hukkinen, Susanna Kauhanen

**Affiliations:** Helsinki, Finland; From the Departments of 1 Plastic Surgery; 2 Radiology, University of Helsinki and Helsinki University Hospital.

## Abstract

**Background:**

Lipofilling can be used to reconstruct a breast without additional implants or autologous composite grafts. However, methods to maximize retention of the transferred fat remain under debate. Here, the authors present their experience of breast reconstruction with lipofilling without concomitant use of tissue expanders.

**Methods:**

Patients who had completed breast reconstruction with lipofilling between June of 2010 and June of 2016 were reviewed. Those with obtainable follow-up magnetic resonance imaging scans were included in this cross-sectional study. The hospital records were reviewed for details of the lipofilling operations. Magnetic resonance imaging scans were evaluated for the volume retention and quality of the transferred fat. The patients were asked to assess the appearance and sensitivity of the reconstructed breast, the recovery time, and any adverse effects at the fat donor area.

**Results:**

Thirty-eight women with 41 reconstructed breasts were included in the study. The median age at follow-up was 62 years (range, 48 to 78 years). They had undergone a median of four (range, two to six) lipofilling procedures with a median total volume 690 mL (range, 369 to 1350 mL). After a median follow-up of 2.1 years (range, 0.4 to 6.8 years), the median proportion of transferred fat retained was 58% (range, 14% to 119%), representing a reconstructed breast volume of 76% (range, 17% to 100%) of the contralateral breast. Oil cysts larger than 10 mm were detected in 7%. Most patients reported being satisfied with the reconstructed breast and experienced few side effects.

**Conclusions:**

Breast reconstruction with lipofilling can be performed with an acceptable number of procedures and no preoperative skin expansion. It extends the option of autologous breast reconstruction to women unsuited for major reconstructive procedures.

**CLINICAL QUESTION/LEVEL OF EVIDENCE:**

Therapeutic, IV.

Breast cancer is the most common cancer in women.^1^ Although the majority of tumors can be treated with breast-conserving surgery in conjunction with adjuvant treatment, locally advanced or extensive disease may necessitate a mastectomy. The option of a mastectomy can also be offered as a risk-reducing procedure to women at genetically increased risk of developing breast cancer. A breast reconstruction following mastectomy, in turn, is associated with a better health-related quality of life.^2–4^ Women who have undergone breast reconstruction with autologous tissues have been found to report greater satisfaction with their breasts and better psychosocial well-being than those with implant-based reconstruction.^5^ Thus, the development of an array of breast reconstruction techniques to suit women of varied requirements can be seen as a priority.

Lipofilling of the breast was first introduced as a method of breast augmentation and as an adjunct to other autologous or implant-based methods of breast reconstruction.^6–9^ In breast reconstruction, lipofilling can be described as the construction of a breast mound with a three-dimensional scaffold composed of thin arrays of fat strands.^10,11^ Most women are suited for breast reconstruction with this technique, as the operation is less invasive than autologous flap reconstruction and involves both a shorter operating time and an easier recovery period. The ideal candidate has ample adipose tissue in multiple areas suitable for fat harvesting and supple skin on the chest.

Early reports incorporated expansion of the skin envelope before and after lipofilling with the Brava external expander device.^12^ The device can, however, be perceived as cumbersome.^13,14^ Immediate and delayed breast reconstruction with lipofilling without any skin expansion with internal or external devices has since been described.^11,15,16^ To date, no clear consensus exists for the best preparation of the fat recipient site, method of fat harvest, or the preparation of the lipoaspirate for infiltration.^17^

Here, we review our experience with 38 patients with 41 breasts reconstructed with lipofilling without any preceding skin envelope preparation and without centrifugation of the lipoaspirate. We assess data on the follow-up magnetic resonance imaging (MRI) scans of the reconstructed breasts to evaluate the quality and volume of the fat tissue produced through lipofilling. We also include patient-reported outcomes measures that suggest high satisfaction with the reconstructed breast.

## PATIENTS AND METHODS

Women who had undergone mastectomy for breast cancer and delayed breast reconstruction with lipofilling in Jorvi Hospital, a subunit of Helsinki University Central Hospital, between June of 2010 and June of 2016 were reviewed. Patients who had completed breast reconstruction with lipofilling alone or lipofilling augmented with a simple local skin and subcutaneous tissue flap were included.

All patients were phoned to inform them about the study and to seek oral consent for participation. Patients who had not undergone breast MRI as a part of their cancer follow-up were invited to undergo MRI of the breasts for the purpose of this study. Only patients for whom follow-up MRI scans were available or obtained were included in the study.

Hospital records were reviewed for the patient’s demographic data, comorbidities, adjuvant treatments, operative details, and follow-up breast imaging findings. The study was approved by Helsinki University Musculoskeletal and Plastic Surgery Research Board and was carried out in accordance with the Declaration of Helsinki.^18^ The report was constructed following the Strengthening the Reporting of Observational Studies in Epidemiology statement.^19^

### MRI and Analysis

The imaging was performed with 3.0 T, with four- or 18-channel coils. Imaging sequences included axial precontrast T1-weighted three-dimensional images and dynamic contrast-enhanced acquisition. All deviant imaging findings in MRI, mammograms, and ultrasound were recorded. Palpable masses or suspicious findings requiring biopsy were recorded.

Three-dimensional volume measurements of the breasts were performed with T1 Flash three-dimensional precontrast sequence lasting approximately 1.6 minutes and analyzed by a breast radiologist using the syngo.via imaging software system.^20^ To enable volume calculation, the breast margins were drawn using free-hand multiplanar reformation technique in multiple slices, after which the software supplemented the missing areas and calculated the total volume. The thickness of the subcutaneous tissue was evaluated in computed tomographic scans, MRI, or ultrasound images taken before the first fat grafting to help define the area created with the lipofilling procedures. In patients who had a local flap performed as a part of their breast reconstruction, volume of the flap was measured separately to enable calculation of the reconstructed breast volume without the flap. The volume of the contralateral breast was measured for reference.

### Patient-Reported Outcomes

A questionnaire addressing aspects of the patient experience was constructed for the purposes of this study. The questions addressed the following: (1) size, shape, consistency, and sensitivity of the reconstructed breast in comparison with the other breast; (2) the difficulty of the recovery time; and (3) pain, bruising, and contour irregularities in the fat donor area at 2 months postoperatively. All questions were answered on a scale from 1 to 10, with 1 representing the worst outcome and 10 representing the best. The questionnaires were mailed to the patients. A prepaid return envelope was provided.

A composite score was constructed for the physical aspects of the reconstructed breast by adding the scores for the questions addressing the size, shape, and consistency of the breast. Similarly, a composite score for the donor area at 2 months was constructed with the responses to the questions addressing pain, bruising, and contour irregularities in the area.

### Statistical Analysis

All numerical data were processed using SPSS.^21^ The data are presented as median (range) unless stated otherwise. The volume measurements are presented as the percentage of the grafted fat retained and as a percentage of the contralateral breast volume.

## RESULTS

### Study Population

**Fig. 1.**
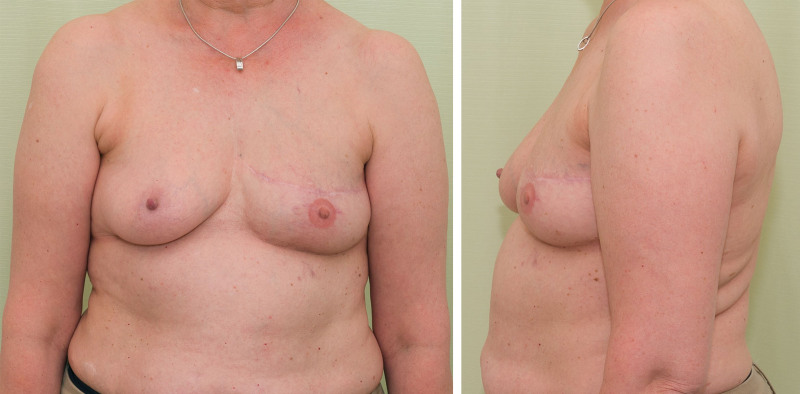
Postoperative photographs of a 53-year-old woman who underwent breast reconstruction with lipofilling. A total of 670 mL of fat was transferred in three operations. MRI volumetric analysis revealed 70% fat volume retention.

**Fig. 2.**
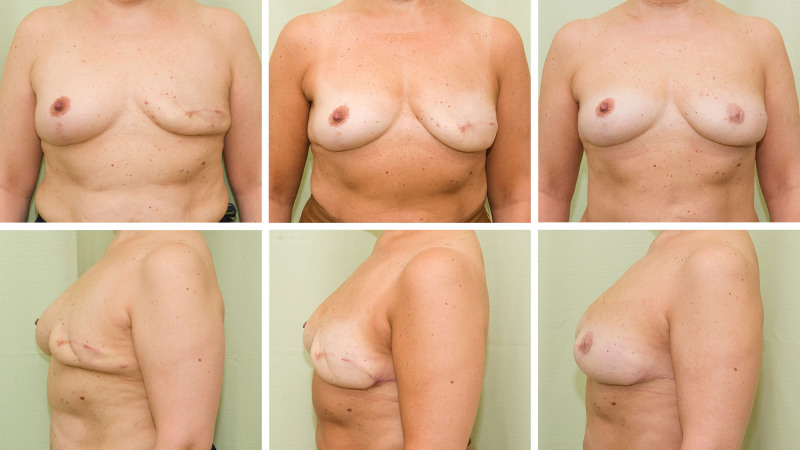
Patient 2 demonstrating breast reconstruction using lipofilling without the use of an expander or an implant. (*Left, above and below*) After two rounds of lipofilling. (*Center, above and below*) After three rounds of lipofilling and a lateral transposition flap. (*Right, above and below*) Three years after the fourth lipofilling and mamilla reconstruction. Total transferred fat volume was 660 mL, of which 89% was retained as assessed with MRI volumetric tissue analysis. The postoperative images are reproduced with permission from Kauhanen S, Höckerstedt A. Full breast reconstruction with fat and how to recycle the “dog-ear.” *Gland Surg*. 2019;8:S297–S300. Photographs of this patient before and after mastectomy, before the first lipofilling operation, are shown in **Figure, Supplemental Digital Content 1**, http://links.lww.com/PRS/F998.

A total of 46 women who had undergone delayed breast reconstruction with lipofilling were identified. Of these, 38 (92%) attended the follow-up MRI and were included in the study. The median age was 62 years (range, 48 to 78 years). Three of the patients had undergone breast reconstruction with lipofilling on both breasts. Thus, a total of 41 reconstructed breasts were analyzed (Table 1). Photographs of a representative postoperative result are shown in Figures 1 and 2. The preoperative images of the woman in Figure 2 are shown. [**See Figure, Supplemental Digital Content 1**, which shows patient 2 in Fig. 2, preoperatively (*left*) and after the mastectomy and contralateral reduction mammaplasty (*right*), http://links.lww.com/PRS/F998.]

**Table 1.** Details of the Patients Who Underwent Breast Reconstruction with Lipofilling^a^

Characteristic	Value (%)
No. of patients	38 (100)
Age at first lipofilling, yr	
Median	58
Range	43–75
BMI, kg/m^2^	
Median	25
Range	18–36
Smoker	2 (5)
Unilateral reconstruction	35 (92)
Bilateral reconstruction	3 (8)
Comorbidities	
None	15 (37)
Hypertension	10 (24)
Type 2 diabetes	6 (15)
Other^b^	21 (51)
No previous adjuvant radiotherapy	
Yes	14 (34)
No	27 (66)
Previous failed reconstruction	3 (7)

BMI, body mass index.

a Data available for 33 patients, assessed preoperatively.

b Other comorbidities included arthrosis, asthma, hypothyroidism, and atrial fibrillation.

Breast cancer was the reason for the mastectomy in all the participants. However, for one of the women with bilateral reconstructions, the second mastectomy was performed as a risk-reducing operation with sparing of the nipple-areola complex, and the first round of lipofilling was performed in the same operation. None of the other mastectomies were skin or nipple-areola complex sparing. The median time from mastectomy to the first lipofilling was 3.5 years (range, 0.0 to 17 years). The median age at the first lipofilling was 58 years (range, 43 to 75 years).

### Fat Grafting Details

The lipofilling procedure used here has been previously described in detail.^10^ The donor site for fat grafting was chosen individually for each patient, based on the distribution of available fat and the patient preference. The most common sites used were the abdomen, the flanks, and the thighs. In general, only one donor site was used for each operation to leave the other areas unscarred for later rounds.

All fat grafting procedures were performed with water jet–assisted liposuction technique using the body-jet system (Human Med, Eclipse Ltd., Dallas, TX). The fat was separated from the fluids in a LipoCollector. Lipoaspirate was drawn into 50-mL syringes, held upright for decantation, and the separated fat was then transferred to 10-mL syringes.

The fat grafting was performed with 10-mL Luer-lock syringes connected to a Cytori cell brush (Cytori Therapeutics, Inc., San Diego, CA). Multiple retrograde passes were performed to the intramuscular, subcutaneous, and subdermal planes from four to six predominantly inferolateral entrance points creating a three-dimensional framework of fat.

### Breast Reconstruction Outcomes

**Fig. 3.**
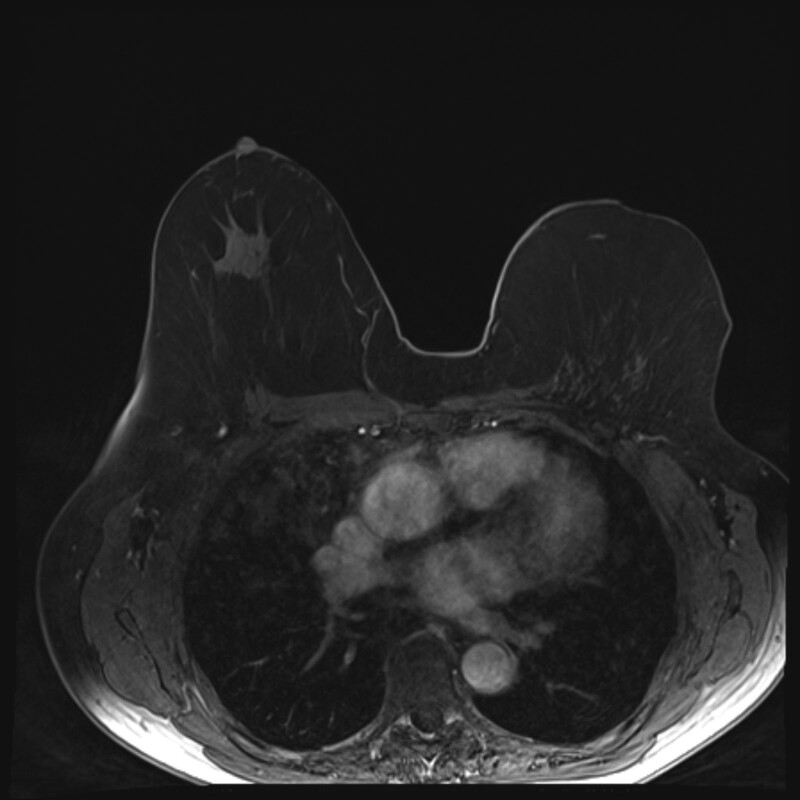
A T1-weighted MRI scan of the breasts of patient 2 from Figure 2, 8 years after the reconstruction of the left breast with four lipofilling operations.

A median of four (range, two to six) lipofilling procedures were performed with a median volume of 200 mL (range, 65 to 440 mL) of fat transferred per procedure (Table 2). No molding of the skin envelope with an expander was performed before the lipofilling. After a median follow-up of 2.1 years (range, 0.4 to 6.8 years), the median volume of the retained fat was 470 mL (range, 78 to 1030 mL). This suggests that 58% (range, 14% to 119%) of the transferred fat had been retained, as judged by the MRI volume analysis. The local flaps constituted 15% (range, 5% to 57%) of the breast volume. The resulting reconstructed breasts were a median 76% (range, 17% to 100%) of the volume of the contralateral breast. A representative MRI image is shown in Figure 3.

**Table 2.** Details of the Breast Reconstructions

	Value (%)
Reconstruction method	
Lipofilling	23 (56)
Lipofilling plus local flap	18 (44)
Contralateral operation	
Mastectomy plus reconstruction	3 (8)
Reduction mammaplasty or mastopexy	23 (61)
None	12 (32)
No. of lipofilling operations	
1	0
2	2 (5)
3	17 (41)
4	14 (34)
5	4 (10)
6	2 (5)
Total fat transfer volume, mL	
Median	690
Minimum	369
Maximum	1350
Clavien-Dindo class of perioperative complications	
0	37 (90)
1^a^	2 (5)
2^b^	1 (2)
3a^c^	1 (2)
3b^d^	1
4 or 5	0

a Two postoperative hematomas that did not require any intervention: one at the fat donor area and another one related to the local flap reconstruction.

b Infection of the reconstructed breast requiring antibiotic treatment.

c Partial necrosis of a local flap.

d Hematoma in the contralateral breast following a reduction mammaplasty.

Four of the reconstructions resulted in a Clavien-Dindo class 3a or lower complication (Table 2). One patient experienced postoperative bleeding in the contralateral breast after a reduction mammaplasty and required a reoperation.

### Imaging Findings

Oil cysts were observed in MRI or ultrasound imaging in 17 reconstructed breasts (41%), with large oil cysts greater that 10 mm in diameter in three (7%). A suspicious enhancing mass was observed on MRI in five breasts (12%), leading to a core needle biopsy. The histology was benign for all the biopsy specimens taken. Multiple focal enhancement in MRI and benign calcifications on mammography were detected in 14 breasts (34%) and 10 breasts (24%), respectively.

### Patient-Reported Outcomes

**Fig. 4.**
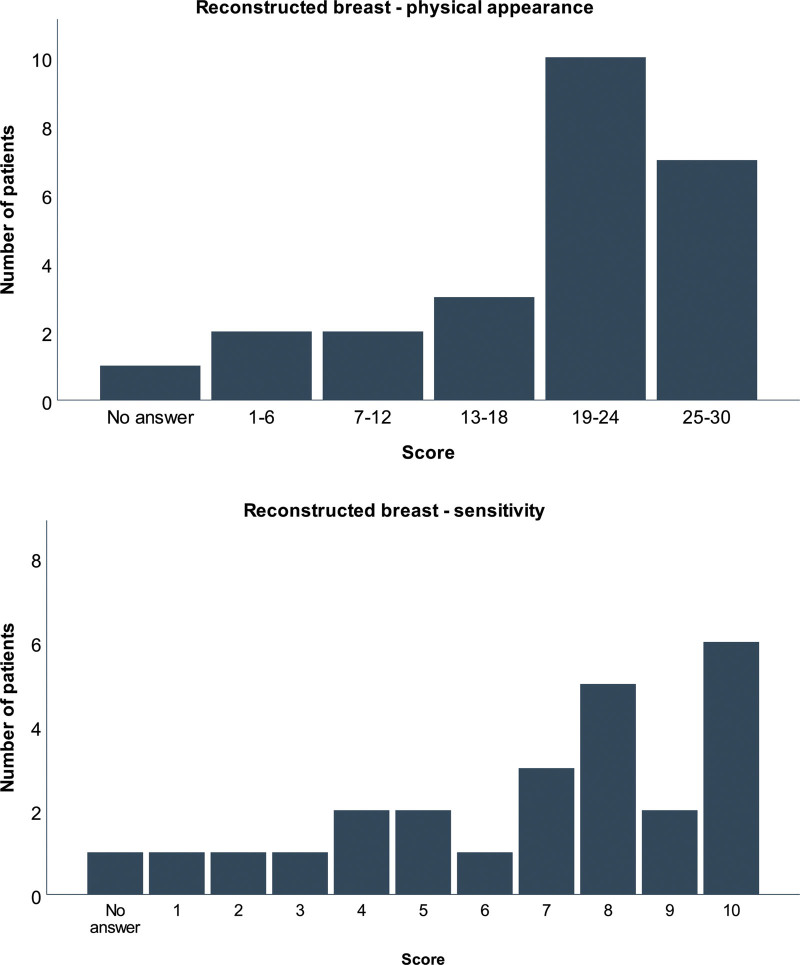
(*Above*) Patient-reported evaluation of the physical aspects of the breast. A score reflecting the patient-perceived similarity of the breast reconstructed with lipofilling and the opposite breast was produced through adding the scores for the size, shape, and consistency, each scored on a range from 1 (very different) to 10 (similar). (*Below*) Patient-reported sensitivity of the breast. Twenty-four patients assigned a score to the sensitivity to touch of their reconstructed breast on a range from 1 (very different from the normal breast) to 10 (similar to the normal breast).

The questionnaire was returned by 25 of the participants (66%). Eleven of the respondents (44%) had a history of adjuvant radiotherapy to the chest. The reported similarity between the physical aspects of the reconstructed breast and the nonoperated breast was high, with a median score of 20 (range, 3 to 28) of the possible 30 (Fig. 4, *above*). The median score for the sensitivity of the breast was 8 (range, 1 to 10) of 10, reflecting a good match with the nonoperated breast (Fig. 4, *below*).

**Fig. 5.**
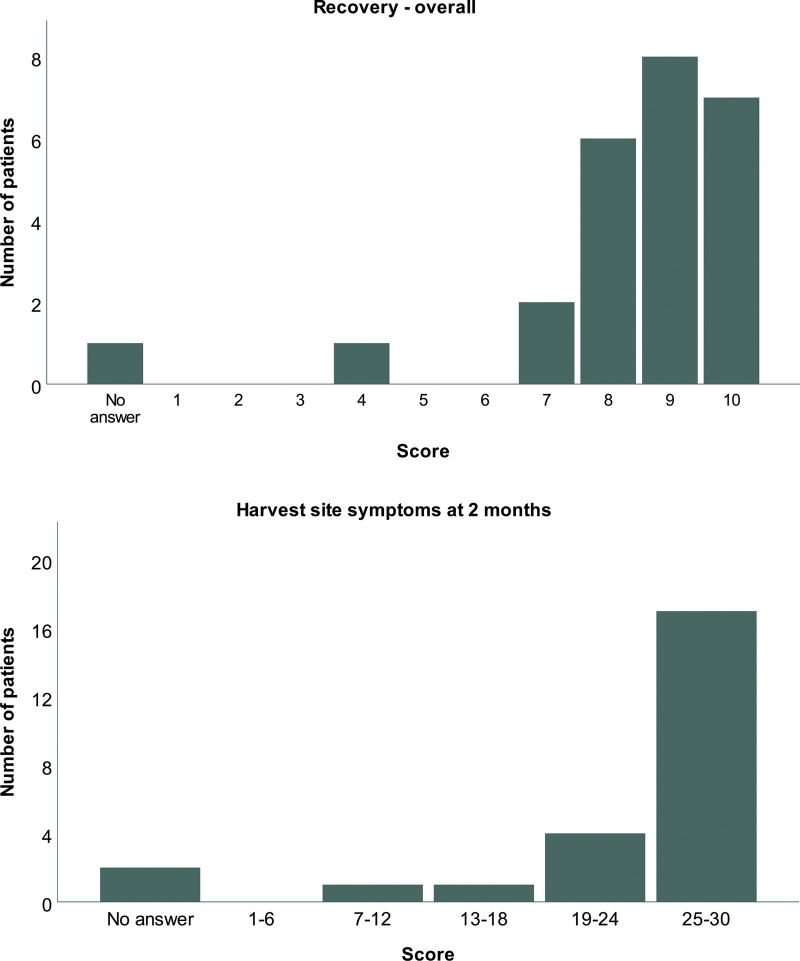
(*Above*) Patient-reported ease of recovery from the lipofilling operation(s). Twenty-four patients scored the recovery from 1 (difficult) to 10 (easy). (*Below*) Patient-reported evaluation of the fat donor area at 2 months after the operation. A composite score was produced through adding the scores for the amount of bruising, pain, and contour irregularities, each scored on a range from 1 (a lot of) to 10 (none at all).

The median score for the ease of recovery was 9 (range, 4 to 10) of 10, suggesting that most of the patients found the recovery period after the lipofilling operations easy (Fig. 5, *above*). Most patients reported little or no pain, bruising, or contour irregularities in the fat donor area at 2 months after the surgery, producing a median score of 29 of the possible 30 (range, 9 to 30) for the recovery of the fat donor area (Fig. 5, *below*).

## DISCUSSION

**Fig. 6.**
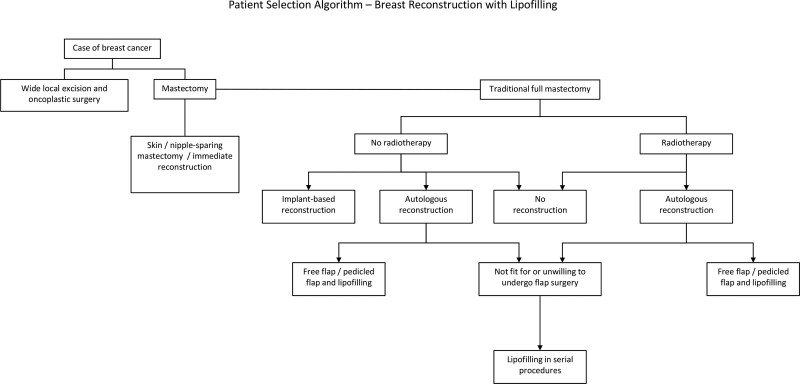
Patient selection algorithm for breast reconstruction with lipofilling.

Lipofilling, or fat grafting, is a relatively new technique for total breast reconstruction following mastectomy, and many still question the reliability of the method.^22^ Here, we presented MRI imaging and patient-reported outcomes data for 38 women with 41 breasts who underwent reconstruction with lipofilling. Although the local skin and subcutaneous tissue excess at the lateral aspect of the mastectomy scar was used to augment the breast as a part of the reconstruction in some of the patients, no implants or pedicled or microvascular flaps were incorporated. Thus, lipofilling is a good option for reconstruction with autologous tissue for women who do not wish, or are not fit for, the more complex reconstructive options (Fig. 6). Overall, we view a paucity of adipose tissue or the inability to attend repeated procedures as the only significant contraindications for this procedure.

No skin expansion device was used before lipofilling during the breast reconstructions. The total volume of fat transferred during the reconstruction process for each patient was 690 mL (range, 369 to 1350 mL). A median of four lipofilling procedures were needed for each breast. This is higher than the average 2.8 procedures for nonirradiated and 4.9 procedures for irradiated mastectomy regions, reported in centers where skin envelope expansion with the Brava device is routinely used.^12^ The total retained volume of the transferred fat was a median of 470 mL, higher than the 375 mL reported with the Brava device.^12^ Interestingly, a recent series including both irradiated and nonirradiated patients demonstrated no impact of Brava use on the number of lipofilling procedures required or the volume of fat transferred per session.^14^ These observations suggest that although the skin expansion may, under some conditions, lead to fewer lipofilling procedures, it is not required for large-volume breast reconstruction with lipofilling.

The median proportion of the transferred fat retained after a median follow-up of 2.1 years was 58%, as judged by MRI volumetric analysis. MRI was chosen here as the imaging modality as, in addition to the good soft-tissue resolution it provides, MRI has been suggested to produce the most reliable results for breast volume estimation.^23,24^ The retention figure is based on the total volume of decanted lipoaspirate injected during the lipofilling operations without accounting for the infiltration fluid contained in the injected fat. Notably, no centrifugation or gauze rolling was performed to reduce the water content of the lipoaspirate.^14,25^ Thus, it is similar to the 78% reported previously for centrifuged lipoaspirate, where approximately 20% of the liquid of the lipoaspirate is discarded before the fat injection.^25^ The fat retention observed in our data are also higher than previously reported after lipofilling used to augment reconstructed or lumpectomy breasts.^26^

The quality of the fat tissue created with lipofilling was high, as judged with MRI scanning. Multiple focal enhancement and small oil cysts under 10 mm in diameter were each detected in 34% of the reconstructed breasts. Larger oil cysts were present in 7%. Benign calcification was detected on mammography in 24% of the breasts. These findings suggest a degree of fat necrosis in the tissue.^27^ A similar figure, with 49% of women with lipofilling of breasts having benign calcification on mammography, has previous been reported in a large series with 670 women.^7^ Overall, our data reflect a high survival of the transferred fat in the recipient site, with a large proportion of women displaying no aberrant findings on imaging. Although most of the women in our series were operated on by two of the authors (S.K. and A.H.), the procedure is simple, reliable, and easy to teach. Good clinical results with lipofilling are achieved in our department by several plastic surgeons and trainees alike.

One third of the mastectomy areas (14 of the 41 reconstructed breasts) had been treated with radiotherapy before lipofilling. This study is thus too small to enable a statistical assessment of the effect of radiotherapy on the survival of the grafted fat. Irradiated breast fields have been suggested to require larger volumes of fat, transferred over more lipofilling procedures, and are associated with greater degree of necrosis in the transferred fat.^11,12,15^ The scarring induced by radiotherapy also increases the amount of rigottomy needed before transplantation.^28^ In contrast, fat transfer has been shown to improve skin quality in an irradiated or scarred area, and is at times used for that sole purpose also in mastectomy areas.^28,29^ Thus, we do not consider irradiation damage in the mastectomy region a contraindication to breast reconstruction with lipofilling, but discuss the possible lower fat take and the subsequent need for a higher number of transfer procedures with the patient.

Adverse effects related to breast reconstruction with lipofilling were rare, with 90% of our patients experiencing no complications perioperatively. Only two patients (4%) required additional surgical procedures, one under local anesthesia for a partial necrosis of a local flap and one under general anesthesia for a hematoma on the contralateral breast after a reduction mammaplasty. One patient required antibiotic treatment for an infection of the reconstructed breast. No fat donor-site infections were reported. Total complication rates between 0% and 5.1% have been reported elsewhere.^11,12,14,15^ A suspicious enhancing mass was detected in the reconstructed tissue with MRI during follow-up in 12% of the participants, leading to core needle biopsies with benign histology. These findings highlight the safety of the lipofilling procedure and its suitability to our patient population that included women up to 75 years old and those with previous failed reconstructions. Notably, the median age of 62 years in our patient population is higher than previously reported for breast reconstruction with lipofilling or other methods.^12,14,30^

The aim of breast reconstruction with lipofilling is to create a breast with acceptable size, shape, and consistency. Our study is, to our knowledge, the first one to present detailed patient-reported outcomes on total breast reconstructions with lipofilling. Most of the patients reported a reasonable similarity in the three physical attributes between their two breasts. Similarly, the sensitivity of the reconstructed breast was reported to be close to normal. MRI volume data were in line with the self-perceived volume symmetry, with the reconstructed breast achieving a median 76% of the contralateral breast volume.

The recovery periods after the lipofilling procedures were reported acceptable overall. In addition, the self-reported donor-site symptoms including pain, bruising, and contour irregularities were rare at 2 months postoperatively. This supports the role of lipofilling as a method of breast reconstruction suitable for those unable or unwilling to undergo a major procedure with a longer operation time and a demanding recovery period. Routinely, most of the procedures are performed on day-surgery bases, with the women able to return to work as early as the first postoperative day. This is a distinct benefit of lipofilling compared with other autologous reconstruction methods. With repeated lipofilling sessions booked 3 months apart, the whole breast reconstruction procedure typically lasts approximately 1 year.

The questionnaires used here were custom-made for this study, as no suitable questionnaires addressing patients’ perception of their breasts were available in Finnish. In addition, no questionnaire focusing on breast reconstruction with lipofilling is, to our knowledge, available internationally. The questionnaires were not validated before use, limiting the strength of the conclusions drawn based on these data.^31^ Furthermore, the questionnaires were completed around the time of the last follow-up, and the evaluation of the postoperative period was performed retrospectively, possibly influencing the results. A prospective study incorporating a validated patient-reported outcomes instrument is needed to fully evaluate the patient experience.

Further limitations to our study include the error introduced to the volume measurement in our study, as the outline of the reconstructed breast was hand-drawn to exclude the estimated subcutaneous tissue. The small local skin and subcutaneous tissue flap, used in 44% of the patients to reshape the lateral aspect of the mastectomy scar, increased the difficulty of defining the tissue created with lipofilling. In addition, evaluation of the oncologic safety of lipofilling after breast cancer is not within the scope of this study.

## CONCLUSIONS

Breast reconstruction with lipofilling can produce a sustainable, natural-appearing, sensate breast with an acceptable number of procedures and no need for preoperative skin expansion. It extends the option of autologous breast reconstruction to women unsuitable for major reconstructive procedures.

## DISCLOSURE

The authors have no financial relationships, activities, interests, or support to disclose.

## ACKNOWLEDGMENT

This research was supported by funding form Helsinki University Musculoskeletal and Plastic Surgery Research Center.

## Supplementary Material



## References

[1] Sung H Ferlay J Siegel RL. Global cancer statistics 2020: GLOBOCAN estimates of incidence and mortality worldwide for 36 cancers in 185 countries. CA Cancer J Clin. 2021;71:209–249. 33538338 10.3322/caac.21660

[2] Eltahir Y Werners LLCH Dreise MM. Quality-of-life outcomes between mastectomy alone and breast reconstruction: comparison of patient-reported BREAST-Q and other health-related quality-of-life measures. Plast Reconstr Surg. 2013;132:201e–209e. 10.1097/PRS.0b013e31829586a7 23897347

[3] Yoon AP Qi J Brown DL. Outcomes of immediate versus delayed breast reconstruction: results of a multicenter prospective study. Breast 2018;37:72–79. 29102781 10.1016/j.breast.2017.10.009 PMC5902735

[4] Howes BHL Watson DI Xu C Fosh B Canepa M Dean NR. Quality of life following total mastectomy with and without reconstruction versus breast-conserving surgery for breast cancer: a case-controlled cohort study. J Plast Reconstr Aesthet Surg. 2016;69:1184–1191. 27406255 10.1016/j.bjps.2016.06.004

[5] Toyserkani NM Jørgensen MG Tabatabaeifar S Damsgaard T Sørensen JA. Autologous versus implant-based breast reconstruction: a systematic review and meta-analysis of Breast-Q patient-reported outcomes. J Plast Reconstr Aesthet Surg. 2020;73:278–285. 31711862 10.1016/j.bjps.2019.09.040

[6] Coleman SR Saboeiro AP. Fat grafting to the breast revisited: safety and efficacy. Plast Reconstr Surg. 2007;119:775–785. 17312477 10.1097/01.prs.0000252001.59162.c9

[7] Illouz YG Sterodimas A. Autologous fat transplantation to the breast: a personal technique with 25 years of experience. Aesthetic Plast Surg. 2009;33:706–715. 19495856 10.1007/s00266-009-9377-1

[8] Zocchi ML Zuliani F. Bicompartmental breast lipostructuring. Aesthetic Plast Surg. 2008;32:313–328. 18188638 10.1007/s00266-007-9089-3

[9] Delay E Garson S Tousson G Sinna R. Fat injection to the breast: technique, results, and indications based on 880 procedures over 10 years. Aesthet Surg J. 2009;29:360–376. 19825464 10.1016/j.asj.2009.08.010

[10] Kauhanen S Höckerstedt A. Full breast reconstruction with fat and how to recycle the “dog-ear.” Gland Surg. 2019;8:S297–S300. 31709171 10.21037/gs.2019.05.07 PMC6819887

[11] Hoppe DL Ueberreiter K Surlemont Y Peltoniemi H Stabile M Kauhanen S. Breast reconstruction de novo by water-jet assisted autologous fat grafting—a retrospective study. Ger Med Sci. 2013;11:Doc17. 24403878 10.3205/000185 PMC3884560

[12] Khouri RK Rigotti G Khouri RKJ. Tissue-engineered breast reconstruction with Brava-assisted fat grafting: a 7-year, 488-patient, multicenter experience. Plast Reconstr Surg. 2015;135:643–658. 25719686 10.1097/PRS.0000000000001039

[13] Khouri RK Rigotti G Cardoso E Khouri RKJ Biggs TM. Megavolume autologous fat transfer: part I. Theory and principles. Plast Reconstr Surg. 2014;133:550–557. 24572848 10.1097/01.prs.0000438044.06387.2a

[14] Zhang X Cai L Yin B Han X Li F. Total breast reconstruction using large-volume condensed and viable fat grafting after mastectomy. J Plast Reconstr Aesthet Surg. 2021;74:966–973. 33341385 10.1016/j.bjps.2020.10.109

[15] Longo B Laporta R Sorotos M Pagnoni M Gentilucci M Santanelli di Pompeo F. Total breast reconstruction using autologous fat grafting following nipple-sparing mastectomy in irradiated and non-irradiated patients. Aesthetic Plast Surg. 2014;38:1101–1108. 25320029 10.1007/s00266-014-0406-3

[16] Serra-Renom JM Muñoz-Olmo J Serra-Mestre JM, Breast reconstruction with fat grafting alone. Ann Plast Surg. 2011;66:598–601. 21508823 10.1097/SAP.0b013e3181f3e33e

[17] Kakagia D Pallua N. Autologous fat grafting: in search of the optimal technique. Surg Innov. 2014;21:327–336. 24480787 10.1177/1553350613518846

[18] World Medical Association. World Medical Association Declaration of Helsinki: ethical principles for medical research involving human subjects. JAMA 2013;310:2191–2194. 24141714 10.1001/jama.2013.281053

[19] Equator Network. Strobe Guideline. Available at: https://www.equator-network.org/reporting-guidelines/strobe/. Accessed October 10, 2021.

[20] syngo.via. Erlangen, Germany: Siemens Healthcare GmbH; 2019.

[21] IBM SPSS Statistics for Windows. Armonk, NY: IBM Corp.; 2017.

[22] Nava MB Blondeel P Botti G. International expert panel consensus on fat grafting of the breast. Plast Reconstr Surg Glob Open 2019;7:e2426–e2426. 31772879 10.1097/GOX.0000000000002426 PMC6846285

[23] Choppin SB Wheat JS Gee M Goyal A, The accuracy of breast volume measurement methods: a systematic review. Breast 2016;28:121–129. 27288864 10.1016/j.breast.2016.05.010

[24] Herold C Ueberreiter K Busche MN Vogt PM. Autologous fat transplantation: volumetric tools for estimation of volume survival. A systematic review. Aesthetic Plast Surg. 2013;37:380–387. 23354764 10.1007/s00266-012-0046-4

[25] Ueberreiter K von Finckenstein JG Cromme F Herold C Tanzella U Vogt PM. BEAULI—a new and easy method for large-volume fat grafts (in German). Handchir Mikrochir Plast Chir. 2010;42:379–385. 21161858 10.1055/s-0030-1267913

[26] Choi M Small K Levovitz C Lee C Fadl A Karp NS., The volumetric analysis of fat graft survival in breast reconstruction. Plast Reconstr Surg. 2013;131:185–191. 23076412 10.1097/PRS.0b013e3182789b13

[27] Fernandes Chala L de Barros N de Camargo Moraes P. Fat necrosis of the breast: mammographic, sonographic, computed tomography, and magnetic resonance imaging findings. Curr Probl Diagn Radiol. 2004;33:106–126. 15215818 10.1067/j.cpradiol.2004.01.001

[28] Rigotti G Marchi A Galiè M. Clinical treatment of radiotherapy tissue damage by lipoaspirate transplant: a healing process mediated by adipose-derived adult stem cells. Plast Reconstr Surg. 2007;119:1409–1422. 17415234 10.1097/01.prs.0000256047.47909.71

[29] Khouri RK Smit JM Cardoso E. Percutaneous aponeurotomy and lipofilling: a regenerative alternative to flap reconstruction? Plast Reconstr Surg. 2013;132:1280–1290. 23924652 10.1097/PRS.0b013e3182a4c3a9

[30] Hamnett KE Subramanian A. Breast reconstruction in older patients: a literature review of the decision-making process. J Plast Reconstr Aesthet Surg. 2016;69:1325–1334. 27498596 10.1016/j.bjps.2016.06.003

[31] Ioannidis JP Greenland S Hlatky MA. Increasing value and reducing waste in research design, conduct, and analysis. Lancet 2014;383:166–175. 24411645 10.1016/S0140-6736(13)62227-8 PMC4697939

