# Supplementary figures and images for: Total Breast Reconstruction with Lipofilling after Traditional Mastectomy without the Use of Tissue Expanders
Source: Plast Reconstr Surg. 2023 Feb 14;152(3):483–91. doi: 10.1097/PRS.0000000000010252 (PMC10461718; doi:10.1097/PRS.0000000000010252)

**a)**

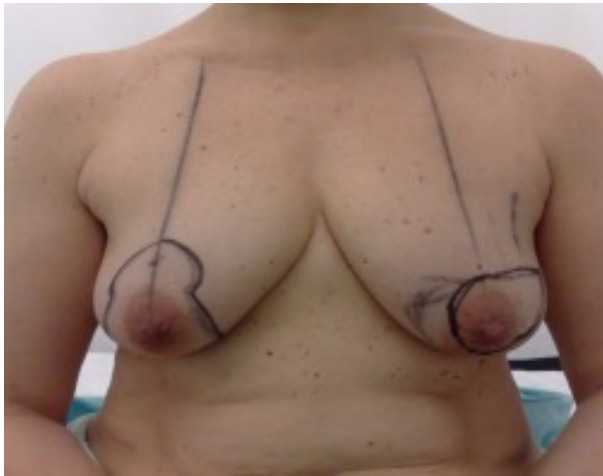

**b)**

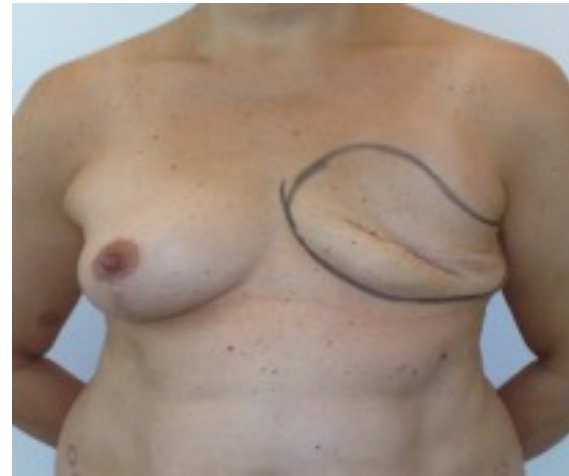

Supplement: Supplementary file 1 [file prs-152-0483-s001.pdf]
